# Prostaglandin and prostaglandin receptors: present and future promising therapeutic targets for pulmonary arterial hypertension

**DOI:** 10.1186/s12931-023-02559-3

**Published:** 2023-11-01

**Authors:** Cheng Zeng, Jing Liu, Xialei Zheng, Xinqun Hu, Yuhu He

**Affiliations:** Department of Cardiology, The Second Xiangya Hospital of Central South University, No.139, Middle Ren-min Road, Changsha, 410011 Hunan Province People’s Republic of China

**Keywords:** Pulmonary Hypertension, Prostaglandin, Prostaglandin receptor

## Abstract

**Background:**

Pulmonary arterial hypertension (PAH), Group 1 pulmonary hypertension (PH), is a type of pulmonary vascular disease characterized by abnormal contraction and remodeling of the pulmonary arterioles, manifested by pulmonary vascular resistance (PVR) and increased pulmonary arterial pressure, eventually leading to right heart failure or even death. The mechanisms involved in this process include inflammation, vascular matrix remodeling, endothelial cell apoptosis and proliferation, vasoconstriction, vascular smooth muscle cell proliferation and hypertrophy. In this study, we review the mechanisms of action of prostaglandins and their receptors in PAH.

**Main body:**

PAH-targeted therapies, such as endothelin receptor antagonists, phosphodiesterase type 5 inhibitors, activators of soluble guanylate cyclase, prostacyclin, and prostacyclin analogs, improve PVR, mean pulmonary arterial pressure, and the six-minute walk distance, cardiac output and exercise capacity and are licensed for patients with PAH; however, they have not been shown to reduce mortality. Current treatments for PAH primarily focus on inhibiting excessive pulmonary vasoconstriction, however, vascular remodeling is recalcitrant to currently available therapies. Lung transplantation remains the definitive treatment for patients with PAH. Therefore, it is imperative to identify novel targets for improving pulmonary vascular remodeling in PAH. Studies have confirmed that prostaglandins and their receptors play important roles in the occurrence and development of PAH through vasoconstriction, vascular smooth muscle cell proliferation and migration, inflammation, and extracellular matrix remodeling.

**Conclusion:**

Prostacyclin and related drugs have been used in the clinical treatment of PAH. Other prostaglandins also have the potential to treat PAH. This review provides ideas for the treatment of PAH and the discovery of new drug targets.

## Background

**Fig. 1:**
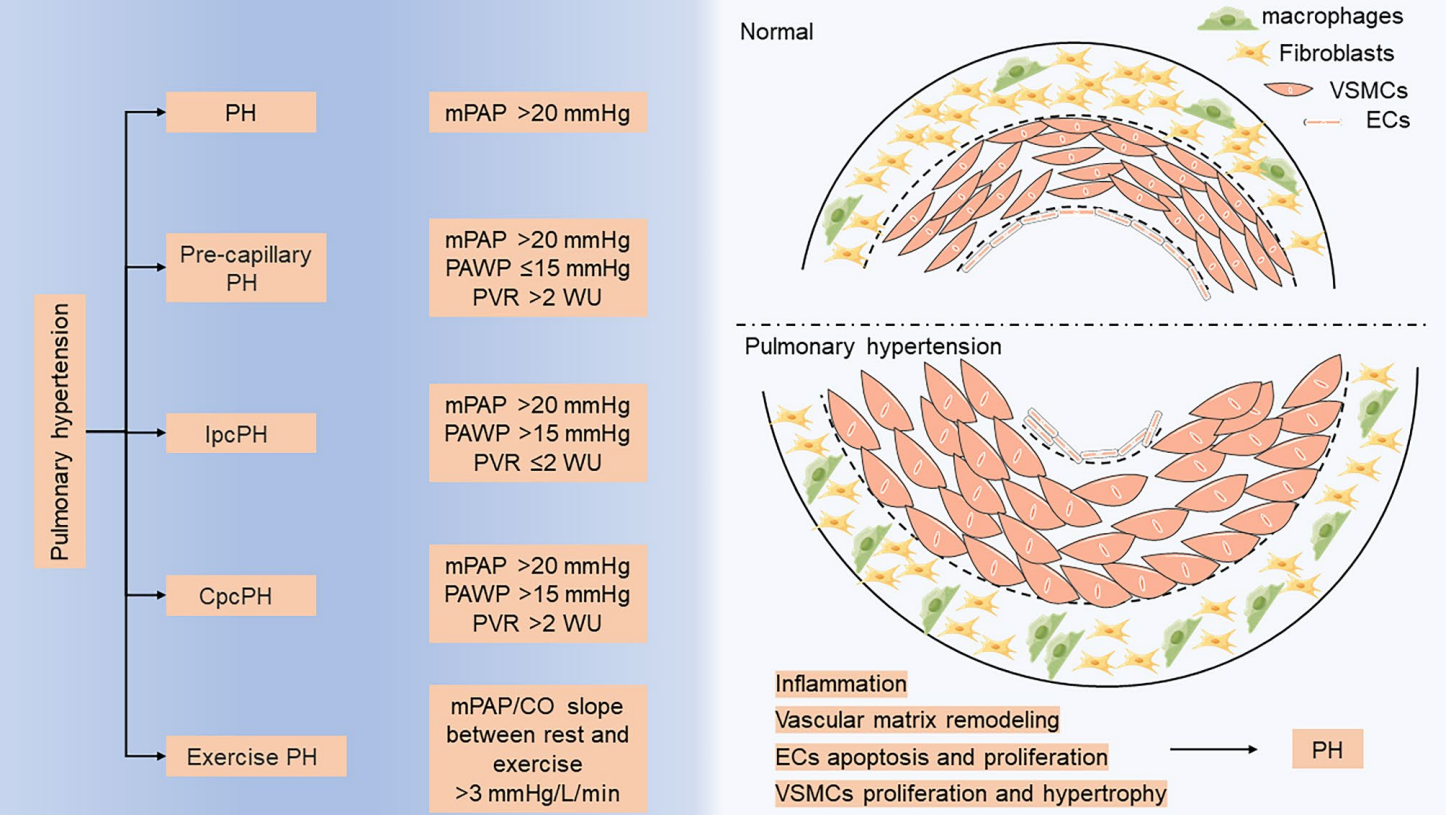
Hemodynamic classification and pathology of PH. The hemodynamics indices of PH include the pre-capillary PH, isolated post-capillary PH, isolated post-capillary PH, and exercise PH. Pathological changes of PH include inflammation, vascular matrix remodeling, EC apoptosis and proliferation, and VSMC proliferation and migration. Abbreviation: CO, cardiac output; Cpc PH, combined post- and pre-capillary pulmonary hypertension; ECs, endothelial cells; Ipc PH, isolated post-capillary pulmonary hypertension; mPAP, mean pulmonary arterial pressure; PAWP, pulmonary arterial wedge pressure; PH, pulmonary hypertension; PVR, pulmonary vascular resistance; VSMC, vascular smooth muscle cells; WU, Wood units

Pulmonary hypertension (PH) is a pathophysiological condition characterized by an abnormal increase in pulmonary arterial pressure caused by a combination of causes that can lead to dyspnea, right heart failure, and even death [1]. The global prevalence of PH is approximately 1%, and it can reach as high as 10% in individuals aged 65 and above as a result of cardiovascular and respiratory factors [2]. Furthermore, right heart failure is present in at least 50% of PH cases [2]. Left heart disease and lung disease are the two leading causes of PH [2]. PH hemodynamics are defined as a mean pulmonary arterial pressure (mPAP) > 20 mmHg measured by the right cardiac catheter at sea level and at resting state [3]. Figure 1 shows in detail the hemodynamic indices of PH, including pre-capillary PH, isolated post-capillary PH, isolated post-capillary PH, and exercise PH [4]. Based on the pathological findings, hemodynamic features, and clinical management strategies, the World Health Organization classified PH into five groups: Group 1, pulmonary arterial hypertension (PAH); Group 2, PH associated with left heart disease; Group 3, PH associated with lung diseases and/or hypoxia; Group 4, PH associated with pulmonary artery obstructions; and Group 5, PH with unclear and/or multifactorial mechanisms [3, 4]. Table 1 provides a detailed description of the classification of PH. This review primarily focused on PAH. The pathophysiological characteristics of PAH include vasoconstriction, extracellular matrix remodeling, and inflammation (Fig. 1). Pulmonary vascular remodeling in PAH is associated with some cellular dysfunction [5]. Abnormal endothelial cells (ECs) apoptosis and proliferation are common pathological features of pulmonary vessels in patients with PAH. Additionally, the proliferation, hypertrophy, and migration of pulmonary artery smooth muscle cells (PASMCs) contribute to severe remodeling of the pulmonary artery, resulting in increased pulmonary arterial pressure (PAP) [6]. The normal interaction between ECs and PASMCs is crucial for maintaining the homeostasis of the lung circulation. Endothelial cells release bioactive agents, including nitric oxide (NO) and endothelin-1 (ET-1), to regulate the function of the underlying smooth muscle cells (SMCs) [7, 8]. Under pathological conditions, the interaction between ECs and SMCs may be changed and certain molecules secreted by endothelial cells exert an influence on the contraction and proliferation of smooth muscle cells. For example, apoptotic ECs release TGFβ1 to induce SMCs proliferation [9] and the endothelial-derived factor CXCL12 induced SMCs proliferation [10].

**Table 1:** Clinical classification of pulmonary hypertension

**Group 1 Pulmonary arterial hypertension (PAH)** 1.1 Idiopathic 1.1.1 Non-responders at vasoreactivity testing 1.1.2 Acute responders at vasoreactivity testing 1.2 Heritable 1.3 Associated with drugs and toxins 1.4 Associated with: 1.4.1 Connective tissue disease 1.4.2 HIV infection 1.4.3 Portal hypertension 1.4.4 Congenital heart disease 1.4.5 Schistosomiasis 1.5 PAH with features of venous/capillary (PVOD/PCH) involvement 1.6 Persistent PH of the newborn
**Group 2 PH associated with left heart disease** 2.1 Heart failure: 2.1.1 with preserved ejection fraction 2.1.2 with reduced or mildly reduced ejection fraction 2.2 Valvular heart disease 2.3 Congenital/acquired cardiovascular conditions leading to post-capillary PH
**Group 3 PH associated with lung diseases and/or hypoxia** 3.1 Obstructive lung disease or emphysema 3.2 Restrictive lung disease 3.3 Lung disease with mixed restrictive/obstructive pattern 3.4 Hypoventilation syndromes 3.5 Hypoxia without lung disease (e.g. high altitude) 3.6 Developmental lung disorders
**Group 4 PH associated with pulmonary artery obstructions** 4.1 Chronic thrombo-embolic PH 4.2 Other pulmonary artery obstructions
**Group 5 PH with unclear and/or multifactorial mechanisms** 5.1 Haematological disorders 5.2 Systemic disorders 5.3 Metabolic disorders 5.4 Chronic renal failure with or without haemodialysis 5.5 Pulmonary tumour thrombotic microangiopathy 5.6 Fibrosing mediastinitis

Some signaling pathways (e.g., NO, endothelin, and prostacyclin pathways) and their modulators have been shown to play key roles in pulmonary vascular tone regulation and remodeling [11, 12]. The overexpression of ET-1 leads to vasoconstriction and vascular cell proliferation [13, 14]. However, NO and prostacyclin (PGI_2_) can lead to vascular dilation and changes in anti-proliferative mechanisms [15]. Currently, drugs targeting the prostacyclin, ET-1, and NO pathways are used in patients with PAH and have been shown to relieve the associated symptoms [16]. Prostacyclin analogs and prostacyclin receptor agonists have shown the potential to enhance exercise capacity, improve quality of life and Borg dyspnea score, and positively impact hemodynamic variables, including mPAP, cardiac index, and pulmonary vascular resistance (PVR) [17]. Endothelin receptor antagonists significantly improve the six-minute walk distance (6MWD), time to clinical worsening, cardiac index, and PVR of patients with PAH [14, 18]. Drugs targeting NO signaling pathway (phosphodiesterase type 5 inhibitors, activators of soluble guanylate cyclase) have demonstrated the potential to improve several clinical parameters, including 6MWD, mPAP, PVR, Borg dyspnea score, and time to clinical worsening [4, 19, 20]. There are five main types of PAH therapeutics: endothelin receptor antagonists, phosphodiesterase type 5 inhibitors, activators of soluble guanylate cyclase, prostacyclin and prostacyclin analogs, and prostacyclin receptor agonists [4]. Current treatments for PAH primarily focus on inhibiting excessive pulmonary vasoconstriction, however, vascular remodeling is recalcitrant to currently available therapies, and lung transplantation remains the definitive treatment for patients with PAH [21, 22]. Therefore, it is imperative to identify novel targets for improving pulmonary vascular remodeling in PAH.

### Prostaglandins

**Fig. 2:**
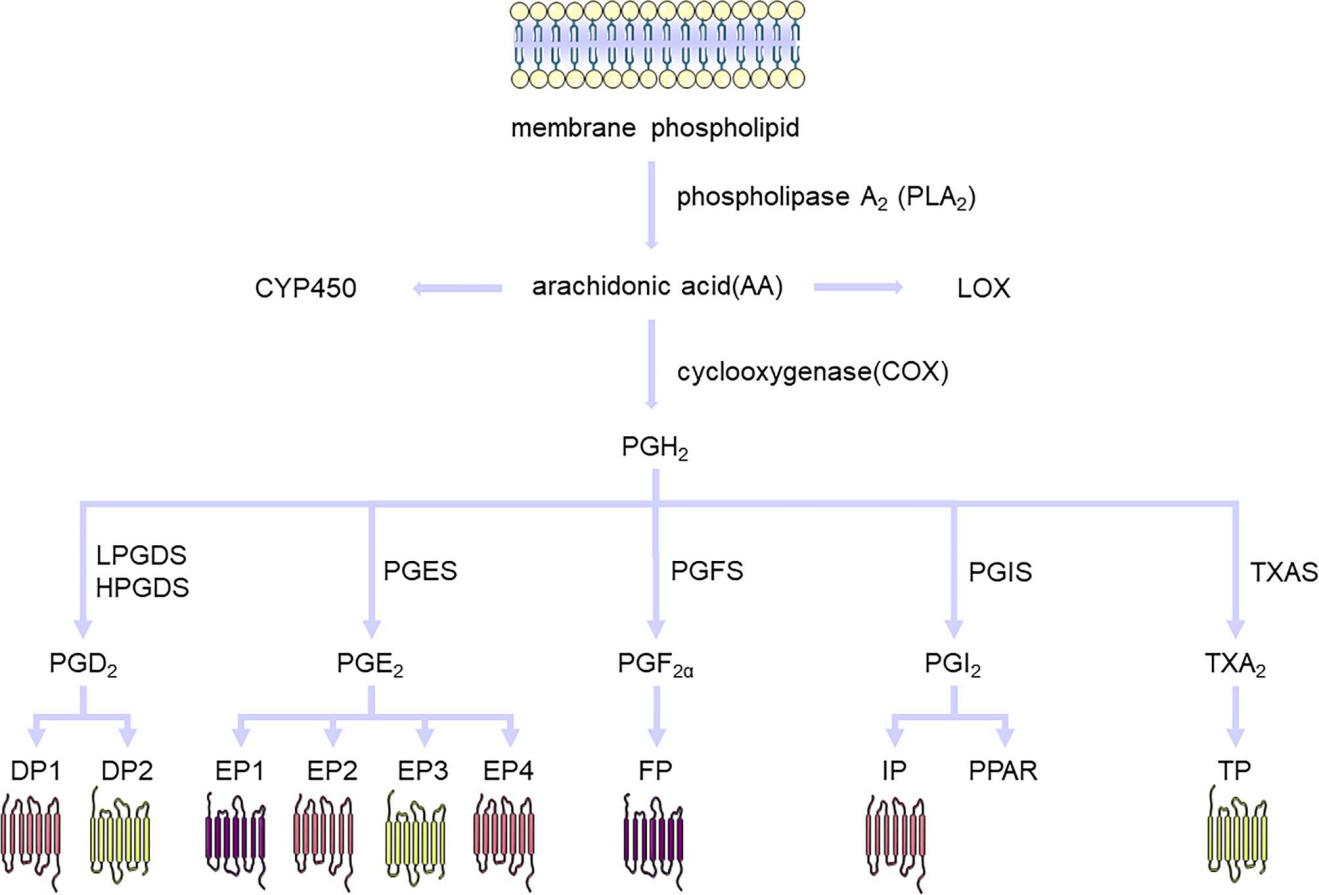
The prostaglandin synthesis pathway and corresponding receptors. AA is released from membrane phospholipids by PLA_2_, and is metabolized to PGH_2_ by COX-1 and COX-2. PGH_2_ is metabolized to TXA_2_ by TXAS, PGI_2_ by PGIS, PGE_2_ by PGES, PGF_2α_ by PGFS, and PGD_2_ by PGDS. TXA_2_ binds to TP, PGI_2_ binds to IP and PPARs, PGE_2_ binds to EPs, PGF_2α_ binds to FP, and PGD_2_ binds to DPs. Abbreviation: AA, arachidonic acid; COX, cyclooxygenase; CYP450, cytochrome P450; LOX, lipoxygenase; PLA_2_, phospholipase A_2_;

In 1935, von Euler preliminarily isolated and extracted PGs from semen and named them PGs [23], which were subsequently successfully isolated [24]. At that time, PGs were thought to be a part of prostate secretion and were eventually found to be produced by seminal vesicles. Subsequently, PGs were found to exist widely in humans and other animals. PGs are a class of lipid-active proteins derived from arachidonic acid (AA), an eicosanoic unsaturated fatty acid. PGs biosynthesis is achieved through three successive enzymatic reactions. First, AA is released from membrane phospholipids by phospholipase A_2_ (PLA_2_) under various physiological and pathological stimuli. Subsequently, under the action of prostaglandin H synthase (PGHS), also known as cyclooxygenase (COX), PGs intermediate metabolites PGG_2_ and PGH_2_ are successively transformed. Finally, prostaglandin terminal synthetases/isomerases including prostaglandin D synthase (PGDS), prostaglandin E synthase (PGES), prostaglandin F synthase (PGFS), prostaglandin I synthase (PGIS), and thromboxane A synthase (TXAS) convert PGH_2_ into various bioactive PGs [25]. In mammals, PGs mainly include prostaglandin D_2_ (PGD_2_), prostaglandin E_2_ (PGE_2_), prostaglandin F_2α_ (PGF_2α_), prostaglandin I_2_ (PGI_2_), and thromboxane A_2_ (TXA_2_) (Fig. 2). After synthesis, prostaglandins are transported into the extracellular microenvironment through simple diffusion. Subsequently, they bind to the prostaglandin receptors to perform various physiological functions [26]. Structural differences among PGs result in different biological activities. They are generally autocrine or paracrine factors and their target cells are located near their secretory sites. In some cases, PGs have different or even opposite effects on different tissues, depending on the type of receptors to which they bind. PGs receptors are a subfamily of G-protein-coupled receptors (GPCRs) known as PGD_2_ receptor 1 (DP1), PGD2 receptor 2 (DP2), PGE_2_ receptor 1 (EP1), PGE_2_ receptor 2 (EP2), PGE_2_ receptor 3 (EP3), PGE_2_ receptor 4 (EP4), prostaglandin F receptor (FP), prostacyclin receptor (IP), and TXA_2_ receptor (TP). EP3 and DP2 receptors inhibit cyclic adenosine monophosphate (cAMP) signaling, whereas EP2, EP4, DP1, and IP receptors activate cAMP signaling [27]. EP1, FP, and TP receptors mainly activate protein kinase C (PKC) and Ca^2+^ pathways [27]. The TP and EP3 receptors also activate Rho. EP2 and EP4 receptors also activate the phosphoinositide 3-kinase (PI3K) and β-arretin pathways [28]. PGs, prostaglandin-synthesis-related enzymes, and PGs receptors are associated with inflammation, cancer, and systemic disease [29]. PGD_2_ has been found to induce sleep, elicit allergic responses, inhibit platelet aggregation, and induce relaxation of both vascular and non-vascular smooth muscle [30]. PGE_2_ promotes tumor development [31], regulates blood pressure homeostasis (with activation of EP2 and EP4 receptors decreasing blood pressure, and activation of EP1 and EP3 receptors increasing blood pressure) [32], facilitates tissue repair and regeneration [33], and contributes to inflammation [34]. PGF_2α_ promotes uterine contraction and vasoconstriction [35, 36]. PGI_2_ plays a role in promoting vasodilation and bronchial relaxation, as well as inhibiting platelet aggregation, inflammation, and proliferation [37]. TXA2 promotes platelet aggregation, airway constriction, and arterial contraction [38].

A single-cell study demonstrated that activation of EP3 receptor enhanced adhesion and cytotoxicity of NK cells toward hepatic stellate cells [39], and various molecules involved in cell adhesion and toxicity can significantly impact the progression of PAH [40, 41]. Another single-cell study showed that PGE_2_ treatment inhibited senescence of decidual cells [42], and senescence-related molecules play a crucial role in the pathogenesis of PAH [43]. However, further research is needed to thoroughly investigate the role of PGs in PAH using single-cell analysis.

### Prostaglandins and pulmonary Hypertension

Currently, research on prostaglandins and their receptors in pulmonary hypertension predominantly focuses on animal models. Common animal models of PH are induced by monocrotaline (MCT), chronic hypoxia, and hypoxia/SU5416. MCT can be used to simulate Group 1 PH, while hypoxia can simulate Group 3 PH. Additionally, hypoxia combined with SU5416 can simulate Group 1/3 PH [44]. MCT induces endothelial damage in pulmonary blood vessels, resulting in the narrowing or occlusion of the vascular lumen [45]. In the MCT-induced model of PH, there is an observed increase in apoptosis of endothelial cells and proliferation of PASMCs [46]. Hypoxia is associated with the development of PH in patients with chronic lung diseases, including interstitial lung disease and chronic obstructive pulmonary disease [47]. Hypoxia-induced PH leads to the thickening of the pulmonary artery wall and increased vasoconstriction. However, the hypoxia model often leads to less severe manifestations of PH with weak VSMCs proliferation and obstructive intimal lesions [48]. In our previous study, we observed that deficiency of DP1 promoted the proliferation of PASMCs in the pulmonary blood vessels of rats treated with MCT; however, we did not observe a significant increase in PASMC proliferation in DP1 knockout mice treated with hypoxia [49]. This finding implies that compared with the hypoxia model, the MCT model is more helpful to observe the anti-proliferation effect of DP1 in vascular remodeling. The hypoxia model does not accurately reflect the same degree of pathological changes observed in patients with PAH, thus further development is needed. Sugen 5416, an antagonist of the VEGF receptor-2 (VEGFR-2), can induce apoptosis in endothelial cells and proliferation in SMCs [50]. The combination of hypoxia and Sugen 5416 leads to severe and progressive remodeling of the pulmonary vasculature, providing a more accurate simulation of Group 1 PH [51]. The more severe PH phenotype animal model makes it more conducive to studying the therapeutic effects of molecules and drugs, such as PGs, in PAH.

**Fig. 3:**
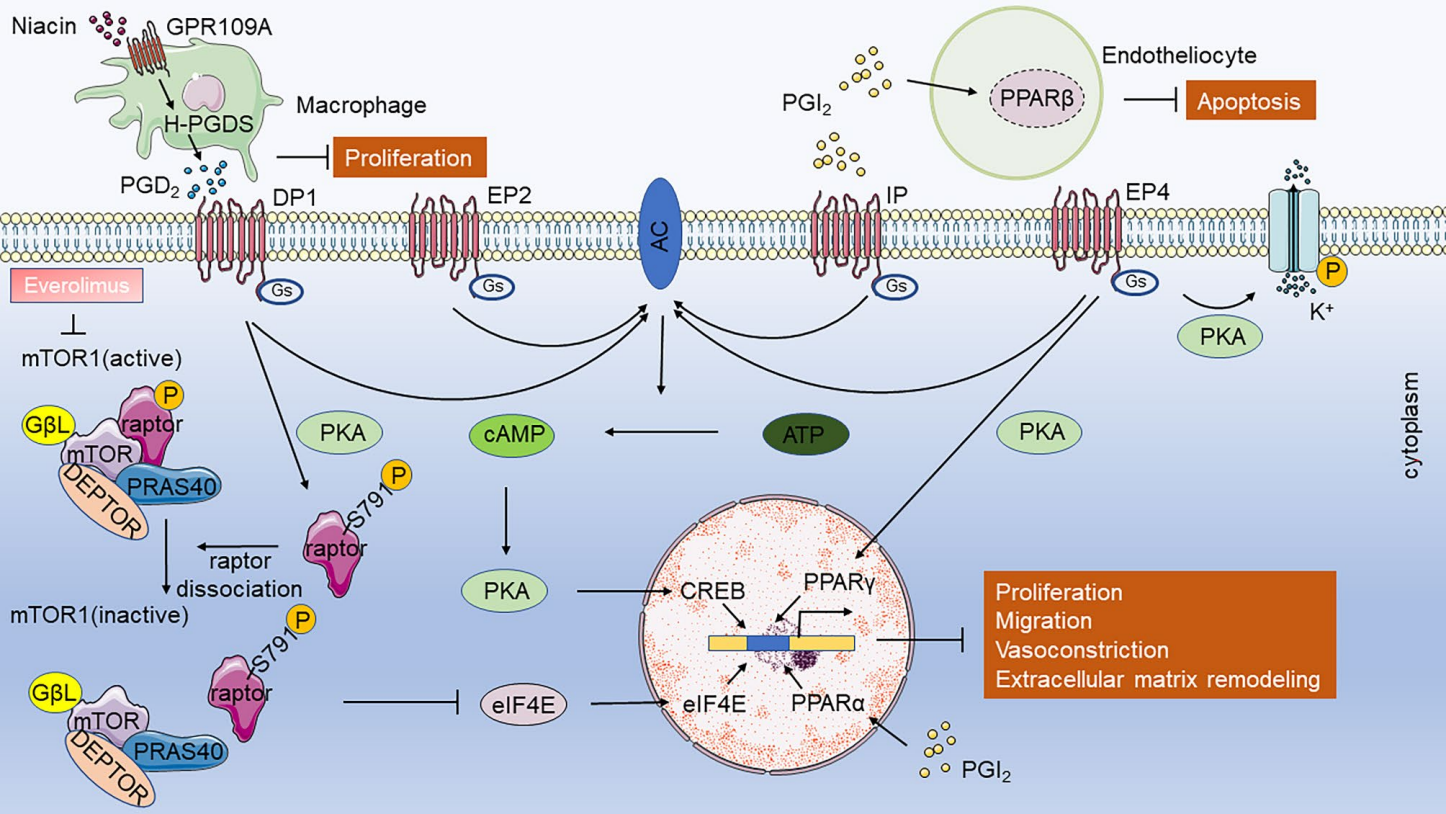
Prostaglandin receptor-related pathways in alleviating pulmonary hypertension. Activation of DP1, EP2, EP4, and IP promotes vasodilation and inhibits the proliferation of pulmonary vascular smooth muscle cells (PVSMCs) through the AC/cAMP/PKA pathway. DP1 activation also attenuates hypertrophy of PVSMCs through PKA-mediated dissociation of raptor from the mTORC1 complex. EP4 also inhibits PVSMC proliferation and migration through PKA/PPARγ and Kv channels. Niacin stimulates the expression of H-PGDS in macrophages and increases the release of PGD_2_. PGI_2_ plays an anti-apoptotic role through PPARβ in endothelial cells and PPARα in VSMCs. Abbreviation: AC, adenylate cyclase; ATP, adenosine triphosphate; cAMP, cyclic adenosine monophosphate; CREB, cAMP-response element binding protein; PKA, protein kinase A; PPAR, peroxisome proliferator-activated receptor

**Fig. 4:**
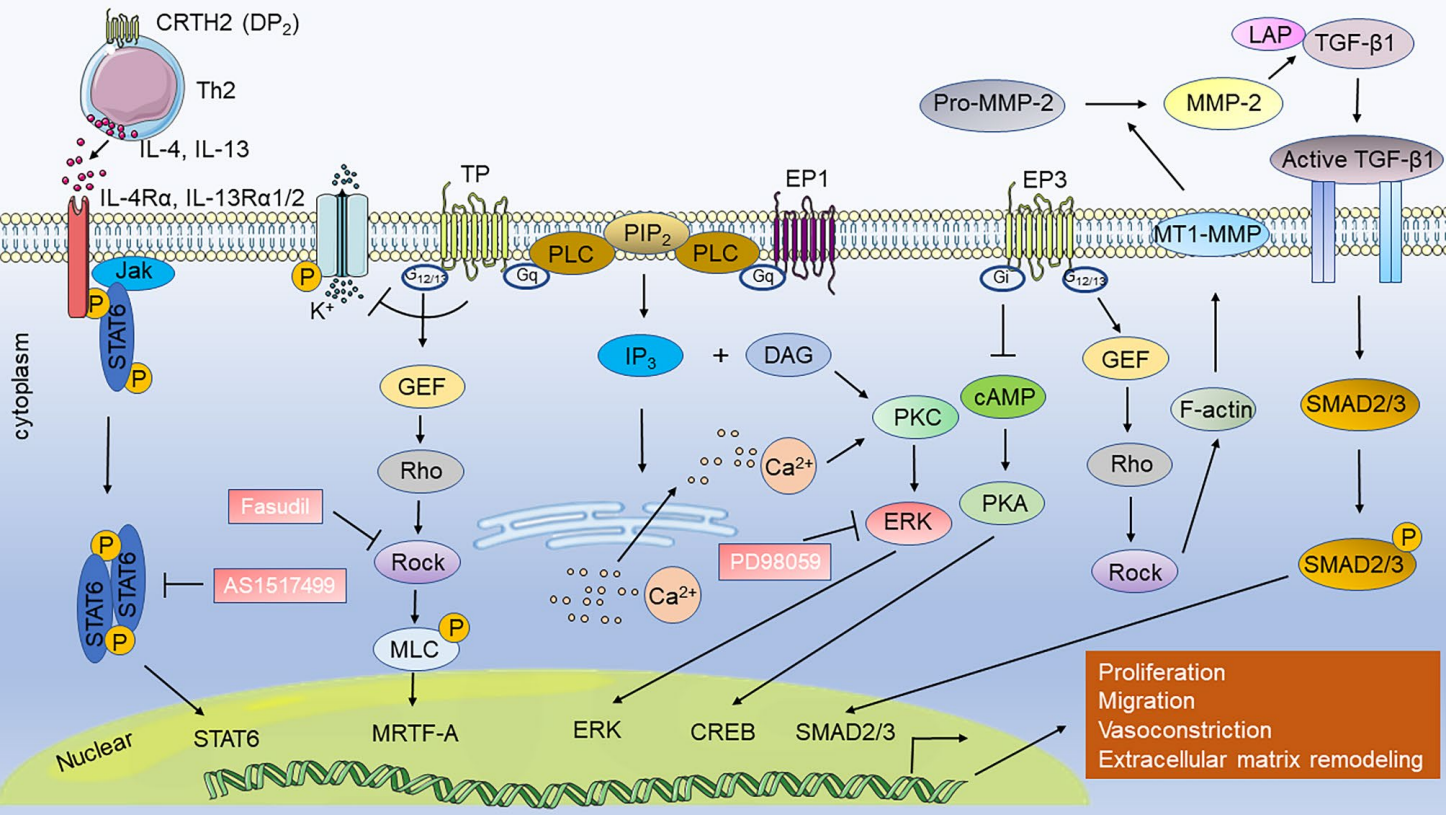
How prostaglandin receptor-related pathways are involved in aggravating pulmonary hypertension. Activation of TP and EP1 promotes vasoconstriction through the PLC/PKC pathway. Via the Rho pathway, EP3 leads to extracellular matrix remodeling and TP leads to vasoconstriction. EP3 can inhibit the cAMP/PKA pathway and TP can inhibit Kv channels. CRTH2 activation in Th2 cells promotes PASMC proliferation by activating STAT6. Abbreviation: CREB, cAMP-response element binding protein; DAG, diacylglycerol; ERK: extracellular signal-regulated kinase; GEF, guanine nucleotide exchange factor; IP3, inositol triphosphate; Jak, Janus kinase; LAP: latency-associated protein; MLC: myosin light chain; MMP, Matrix metalloproteinase; MRTF-A, myocardin-related transcription factor A; MT1-MMP, membrane type 1-matrix metalloproteinase; PIP2, phosphatidylinositol (4,5) bisphosphate; PKC, protein kinase C; PLC, phospholipase C; Rock, Rho-associated protein kinase; SMAD, small mother against decapentaplegic; STAT6, signal transducer and activator of transcription 6; TGF-β1: transforming growth factor beta

AA causes vascular contraction, phenotypic transformation of SMCs, and an imbalance in endothelial cell proliferation and apoptosis, mainly through various derivatives including PGH_2_, PGE_2_, TXA_2_, 12-HETE (12-hydroxy-5,8,10,14-eicosatetraenoic acid), 15-HETE, LTB4 (leukotriene B4), epoxyeicosatrienoic acids (EETs), ultimately leading to vascular remodeling [52–54]. AA has three metabolic pathways: COX, lipoxygenase (LOX), and cytochrome P450 (CYP450). There are two isoforms of cyclooxygenase: COX-1 and COX-2. COX-1 is constitutively expressed in the majority of tissues, whereas COX-2 is constitutively expressed at lower levels but is induced in inflammation and hypoxia [55]. COX-2 plays a role in cardiovascular diseases, including changes in PH [53]. The COX-2 protein is associated with PH in multiple species. COX-2 is overexpressed in the lung tissues of children with PH, increased in hypoxia-induced human pulmonary artery smooth muscle cells (HPASMCs) in vitro, and has an anti-proliferative function [56, 57]. In rats, a significant increase in COX-2 expression in pulmonary vessels and SMCs was observed after hypoxia induction; however, COX-1 expression did not significantly change. Moreover, SC236, a selective COX-2 inhibitor, aggravated PH [58]. COX-2-dependent contractile factors caused abnormal pulmonary artery responses in piglets exposed to hypoxia for three days [59]. In mice, both genetic deletion of COX-2 and the pharmacological inhibition of COX-2 by nimesulide exacerbated hypoxia-induced PH by acting on vascular remodeling, specifically characterized by PASMCs hypertrophy, without inducing cell proliferation [60]. In addition, in vitro experiments have demonstrated that COX-2 deficiency enhances the contractility of hypoxia-induced vascular SMCs and their interactions with the extracellular matrix [60]. In addition to its role in hypoxia-induced PH, COX-2 plays a role in MCT-induced PAH. In MCT-induced PAH mouse models, COX-2 knockdown exacerbates oxidative stress-derived endothelial dysfunction, vasoconstriction, and mild inflammation, thereby aggravating PAH [61]. Bone marrow-derived endothelial progenitor cells (BMEPCs) effectively attenuated MCT-induced PAH in rat models, and the protective effects of BMEPCs on pulmonary vessels may be mediated by the COX-2/PGI_2_/cAMP pathway [62]. Although COX-1 expression in PH lung tissue did not change significantly, endotracheal administration of COX-1 alleviated MCT-induced PAH and right ventricular hypertrophy in rats [63]. Drugs that target COX-2 have side effects because there are numerous downstream molecules of COX-2, such as prostaglandin and prostaglandin receptors. Drugs that target downstream molecules have improved safety and efficacy [53]. Prostacyclin, prostacyclin analogs and receptor agonists, including selexipag, epoprostenol, beraprost, iloprost, and treprostinil, have been clinically used to treat PAH. Prostacyclin analogs exhibited heterogeneous binding affinities to other PG receptors, which can result in varying clinical efficacy [64, 65]. Prostacyclin and prostacyclin analogs do not act solely on IP receptor. Epoprostenol mainly acts on IP and EP3; beraprost mainly acts on IP; iloprost mainly act on IP, EP1and EP3; and treprostinil mainly acts on IP, EP2, and DP1. (Tables 2 and 3). DP1, EP2, EP4, and IP signaling pathways mainly improve pulmonary vascular remodeling, thus improving PAH (Fig. 3), while DP2, EP1, EP3, and TP signaling pathways aggravate PAH (Fig. 4). Compared to iloprost, treprostinil demonstrated a more sustained effect on PVR and exhibited better tolerance, due to its differential affinity for specific prostaglandin receptors [66]. Various prostaglandin receptors play distinct roles in the development and progression of PH. Therefore, in this review, we summarized the progress of prostaglandins and prostaglandin receptors in the study of PAH.

**Table 2:** Therapeutic indications, administration, side effects and corresponding receptors of prostacyclins

Drugs	Therapeutic indication	Administration	Side effects	Prostaglandin receptors
Prostacyclin and prostacyclin analogs				
Epoprostenol	PAH	Intravenous	Headache, gastrointestinal symptoms, infusion site infection	IP, EP1, EP3, TP
Iloprost	PAH	Inhalation	Headache, low blood pressure	IP, EP1, EP2, EP3, EP4, DP1, FP, TP
Treprostinil	PAH	Intravenous, subcutaneous, inhalation, oral	Headache, gastrointestinal symptoms, pain at the infusion site	IP, EP1, EP2, EP3, EP4, DP1, FP
Beraprost	PAH (Japan, South Korea)	Oral	Headache, gastrointestinal symptoms	IP, EP3, EP4
Prostacyclin receptor agonist				
Selexipag	PAH	Oral	Headache, gastrointestinal symptoms	IP

**Table 3:** Comparison of binding affinity between different prostacyclins and various prostaglandin receptors

Drugs	Receptors								
	IP	EP1	EP2	EP3	EP4	DP1	FP	TP	
Prostacyclin and prostacyclin analogs									
Epoprostenol	++++	++	/	++++	N	/	/	++	
Iloprost	++++	++++	+	+++	++	+	++	+	
Treprostinil	++++	++	++++	+	++	++++	+	N	
Beraprost	+++	N	N	++	+	N	N	N	
Prostacyclin receptor agonist									
Selexipag	++++	N	N	N	N	N	N	N	

“+”: affinity strength. The greater the number, the stronger the affinity. “N”: no affinity. “/“: data not available

## Prostaglandin D_2_ and prostaglandin D_2_ receptors

### Prostaglandin D_2_

There are two distinct types of PGD_2_ synthases: lipocalin-type PGD_2_ (L-PGDS) and hematopoietic PGD_2_ (H-PGDS) [67]. L-PGDS is mainly expressed in endothelial cells and cardiomyocytes of the cardiovascular system [68]. H-PGDS is highly expressed in macrophages and mast cells [69]. Systemic biosynthesis of PGD_2_ occurs mainly via H-PGDS in mice [70]. The physiological function of PGD_2_ varies depending on the cell and tissue type, mainly according to the receptor type to which it binds. PGD_2_ was significantly elevated in patients with primary PH [71]. A large infusion of PGD_2_ specifically reversed induced PH and prevented hypoxic pulmonary vasoconstriction in newborn lambs without changing their systemic blood pressure [72]. However, although PGD_2_ is a specific pulmonary vasodilator in fetuses and newborn animals, it does not reduce pulmonary blood pressure in newborn infants with persistent PH syndrome or improve oxygenation [73]. We have found that niacin prevents the progression of MCT-induced and hypoxia/SU5416-induced PAH in rats and suppresses the development of hypoxia/SU5416-induced PAH in mice by enhancing the expression of H-PGDS in lung tissue macrophages and increasing the release of PGD_2_, which inhibits the hypertrophy of pulmonary vein smooth muscle cells (PVSMCs) and improves the remodeling of pulmonary vessels. Deletion of H-PGDS exacerbated hypoxia/SU5416-induced PAH in mice and eliminated the protective effects of niacin against PAH, but not L-PGDS [74]. However, in this study there was no direct evidence of the mechanisms underlying receptor PGD_2_ function.

### Prostaglandin D_2_ receptor 1

DP1 is a protein encoded by the PTGDR1 gene (also known as PTGDR) located at q22.1 on chromosome 14, a chromosomal site associated with asthma and other allergic diseases [75, 76]. PGD_2_, PGE_2_, PGF_2α_, PGI_2_, and TXA_2_ prostaglandins are endogenous ligands of DP1, of which PGD_2_ has the highest affinity and is also a DP1 ligand in other rodents [64, 65, 77–80]. DP1 is mainly expressed in cells involved in mediating allergic and inflammatory responses, namely mast cells, basophil and eosinophils, Th2 cells, and dendritic cells in humans and rodents, and cells that contribute to these responses, namely human and rodent airway epithelial cells, vascular endothelial cells, and goblet cells [81, 82]. DP1 has been linked to allergic diseases such as rhinitis and asthma [83]. Also, DP1 plays an important role in neurological diseases [84], reproductive development [85], digestive tract diseases [86], cardiovascular diseases [69], and maintaining hemodynamics in rodents and humans, including ischemia-reperfusion injury and niacin induced vasodilation [87, 88]. DP1 is expressed in both the pulmonary artery and veins, and its activation induces vascular relaxation [89]. Treprostinil induces pulmonary venous relaxation in part by acting through DP1, and its effect on DP1 in human pulmonary veins may contribute to the treatment of PAH [90]. Recently, we have been reported that DP1 activation prevents hypoxia-induced PH through PKA/raptor-dependent mTORC1 (mammalian target of rapamycin complex 1) dissociation. The DP1 expression level is downregulated in the pulmonary arteries (PAs) of various PAH animal models and patients with idiopathic PAH. Furthermore, DP1 receptor knockout in mice aggravated hypoxia/SU5416-induced PAH by increasing mTORC1 activity; therefore, DP1 activation provides protection against hypoxia-induced PH through PKA/raptor-dependent mTORC1 dissociation. DP1 activation also attenuates hypoxia-induced PA remodeling and hypertrophy of pulmonary VSMCs through the PKA-mediated dissociation of raptor from the mTORC1 complex [49]. This provides direct evidence for the pathogenesis of DP1 in PAH.

### Prostaglandin D_2_ receptor 2

The PTGDR2 gene and GPR44 together code for the protein known as prostaglandin D_2_ receptor 2 (DP2 or CRTH2) [91]. It is selectively expressed in Th2 cells, and is therefore a chemoattractant receptor homologous molecule expressed in Th2 cells (CRTH2) [92, 93]. PTGDR2, located at q12.2 on human chromosome 11, consists of two introns and three exons, and encodes a GPCR composed of 472 amino acids [92]. CRTH2 is also expressed in eosinophils [94], mast cells [95] and group 2 innate lymphoid cells (ILC2s) [96]. PGD_2_ induces chemotaxis of these immune cells through CRTH2 signaling, which is the main pathway driving type 2 inflammation [96–98]. The PGD_2_/CRTH2 signaling axis has been extensively studied in type 2 inflammation-related diseases such as asthma and atopic dermatitis [83, 99]. Therefore, CRTH2 antagonists may develop into a novel type of anti-inflammatory medication [100–103]. The infiltration of inflammatory cells around the pulmonary vessels is usually observed in patients with PH [104], and similar pulmonary inflammation has been observed in experimental PH models [105, 106]. In addition, some patients with immune diseases (such as systemic sclerosis and systemic lupus erythematosus) also develop PH [107–109]. Inflammation and immune disorders may exacerbate PH development, and anti-inflammatory therapy may improve PH development in patients [110]. For instance, acetazolamide prevented PVSMCs dedifferentiation and proliferation in the hypoxia/SU5416 induced rat PAH model through the inhibition of macrophage carbonic anhydrase [111]. Interferon regulatory factor 7 (IRF7) inhibited inflammation by suppressing NF-κBp65 signaling and improved vascular remodeling in MCT-induced rat models of PAH [112]. It has been found that patients with idiopathic PAH and rodents with PAH models exhibit higher levels of CRTH2 expression in their circulating CD4 T cells. CRTH2 knockout significantly improved pulmonary remodeling and PAH in different PAH mouse models through inhibiting Th2 activity via reducing the secretion of IL-4 and IL-13 by Th2 cells [113]. Furthermore, CRTH2 activation in Th2 cells promoted PASMC proliferation by activating signal transducer and the activator of transcription 6 (STAT6) [113].

PGD_2_ primarily exerts its effects through DP1 and DP2 receptors, which exhibit antagonistic roles in PAH. DP1, expressed in PAs and veins, contributes to the improvement of PAH upon activation [89]. Conversely, activation of DP2, predominantly present in Th2 cells, aggravates PAH. Selective activation of DP1 with inhibition of DP2 becomes crucial in the treatment of PAH associated with PGD_2_.

## Prostaglandin E_2_ and prostaglandin E_2_ receptors

### Prostaglandin E_2_

PGE_2_ is produced by various cell types in the body, such as epithelial cells, fibroblasts, and infiltrating inflammatory cells [33] and mediates many physiological and pathological processes, including vascular homeostasis, inflammation, pain, and kidney function [114, 115]. PGE_2_ performs various complex biological functions by binding to different downstream prostaglandin E receptors, including EP1, EP2, EP3, and EP4 [116]. PGE_2_ expression was elevated in a hypoxia/SU5416-induced rat PAH model [117]. PGE_2_ mediates anoxic constriction of the rat intrapulmonary artery (IPA) [118] and may inhibit intense constriction of PASMCs in response to hypoxia in mice [60]. In addition, intravenous infusion of PGE_2_ reduced PAP by reducing cardiac output (CO) in broiler chickens but did not dilate the pulmonary vasculature [119]. Moreover, in samples isolated from human patients, impairment of PGE_2_-induced bronchodilation may be involved in Group 3 PH pathogenesis [120]. The pathway of action of PGE_2_ depends on the cell type and the receptor to which it binds.

### Prostaglandin E_2_ receptor 1

The PTGER1 gene encodes the protein known as EP1, located at the p13.12 locus of human chromosome 19, and contains two introns and three exons and encodes a GPCR [121]. PGE_2_ activates phospholipase C (PLC), which then triggers PKC, increasing phosphatidylinositol hydrolysis and intracellular calcium concentrations through binding to the EP1 receptors [122]. EP1 is widely expressed in rodent tissues and cells including the kidneys, lung, stomach, thalamus, and central nervous system, but is only distributed in a few human organs and cells, such as the pulmonary vasculature, myometrium, and colonic longitudinal muscles [123–127]. To date, animal studies on EP1 have mainly focused on pain, cancer, and renal function with few studies on cardiovascular [34, 128–130]. In terms of cardiovascular function, EP1 plays a major role in blood pressure regulation. PGE_2_ acts on EP1 receptor to cause vascular contraction and increased blood pressure [131]. Gene knockout of the EP1 receptor significantly reduces basal blood pressure and inhibits Ang II-induced hypertension and associated organ damage in mice. Sulprostone, a nonselective agonist of EP1/EP3 receptors, promotes vasoconstriction and increases blood pressure; this vasoconstriction effect is significantly inhibited in EP1/EP3 receptor-knockout mice [132]. However, the role of EP1 in PH has not yet been elucidated. The Th1-mediated immune reaction in vivo is influenced by the PGE_2_- EP1 pathway, which alters the Th1/Th2 balance toward Th1 dominance [133]. The Th1 cytokine IFN-γ is required for the development of the pneumocystis-associated PH mouse model [134]. Therefore, EP1 may affect the development of PH by promoting the differentiation of CD4 T cells toward Th1 cells; however, direct evidence of this mechanism is lacking.

### Prostaglandin E_2_ receptor 2

The human gene PTGER2 encodes EP2, which is the prostaglandin receptor of PGE_2_. It is located at the p22.1 position of human chromosome 14 and contains two introns and three exons, encoding GPCRs [135]. EP2 receptors mediate the increase in cAMP levels and cAMP levels can increase EP2 receptor expression [136]. After binding to PGE_2_, the EP2 receptor initiates the PI3K/protein kinase B (PKB) pathway through the dissociated Gβγ subunit, which phosphorylates and inactivates glycogen synthetase kinase 3 (GSK-3), stabilizing β-catenin and causing nuclear translocation as well as the expression of genes that promote inflammation and growth [137, 138]. The transcription factor nuclear factor κB (NF-κB), which is then transported to the nucleus and mediates the transcription of a range of genes implicated in inflammation, may also be phosphorylated by activated PI3K/PKB [139, 140]. The EP2 receptor prevents neutrophil phospholipase D pathway activation [141] and causes Th1 cell differentiation [142], which is dependent on PI3K. The EP2 receptor is extensively expressed in humans and has been found to be concentrated in the cerebral cortex, articular cartilage, lungs, and smooth muscle, where it influences a number of physiological processes including neural plasticity, immunoregulation, and vasodilation [27, 143]. In addition, PGE_2_/EP2 signaling promotes cell proliferation [140, 144]. A growing number of studies have revealed that EP2 is crucial for PAH. Treprostinil has a high affinity for DP1, EP2, and IP receptors, and part of its effect on PAH therapy is mediated by EP2 receptors [65]. In humans, the majority of inherited PAH cases are linked to mutations in members of the transforming growth factor (TGF) receptor superfamily [89]. The expression of transforming growth factor β1 (TGF-β1) in ECs can promote the differentiation of SMCs into a synthetic phenotype [145]. TGF-β1 plays a crucial role in the development of PAH in animal models. SD-208, TGF-beta receptor I inhibitor, can improve pulmonary vascular remodeling, leading to an amelioration of PH [146, 147]. Therefore, the inhibition of TGF beta 1 is important to prevent pulmonary vascular remodeling and PAH development. The EP2 receptor antagonist PF-04418948 inhibits the expression of TGF-β1 of lung fibroblasts in humans, and enhances the expression of the genes for collagen production (COL1A1 and COL1A2) and fibroblast contractility (ACTG2) when treprostinil is present [147]. By reducing the production and accumulation of type I collagen and fibronectin, treprostinil has a positive preventative effect on pulmonary vascular wall remodeling [148]. PF-04418948 partially or completely reversed treprostinil-induced decline in COL1A1, COL1A2, and ACTG2 expression [147]. In addition, activation of the prostaglandin EP2 receptor inhibits fibroblast functions, including proliferation, migration, and the transition from fibroblasts to myofibroblasts [149]. EP2 is a major subtype of the prostaglandin receptor in PH that inhibits fibrosis and fibrosis-induced remodeling. Moreover, EP2 receptor was upregulated in HPASMC and PAs from PAH patients, suggesting that EP2 may play a role in the development of PH [150]. Butaprost, a highly selective EP2 receptor agonist, was utilized to investigate the potential role of EP2 in PH development. Butaprost administration led to a concentration-dependent decrease in HPASMC proliferation. PF-04418948 abolishes the antiproliferative effects of butaprost. The anti-proliferative effect of the therapeutic dose of treprostinil appears to depend primarily on the activation of the EP2 receptor in HPASMCs in patients with PAH [150]. Therefore, targeting EP2 receptors is a promising strategy for the treatment of PAH.

### Prostaglandin E_2_ receptor 3

The human gene PTGER3 encodes the prostaglandin EP3 receptor, commonly known as EP3. The gene PTGER3 generates GPCRs from the rhodopsin-like receptor family and is found at the p31.1 region of human chromosome 1 [151, 152]. In humans, PTGER3 encodes at least eight different isomers, namely PTGER3-1 to PTGER3-8 (EP3-1, EP3-2, EP3-3, EP3-4, EP3-5, EP3-6, EP3-7, and EP3-8). In mice, PTGER3 encodes at least three isomers, Ptger1-Ptger3 (i.e. Ep3-α, Ep3-β, and Ep3-γ) [81, 153]. These isomers are variations created by selective 5’-end splicing of DNA to create proteins with altered C-termini or regions therein [154, 155] and may perform different functions due to differences in tissue expression and the signaling pathways they activate [156]. EP3 receptor is widely distributed in human tissues and play crucial functions in the kidneys, urinary bladder, reproductive system, brain, and cardiovascular system [157]. Similar to most other prostaglandin receptors, the EP3 receptor has been involved in cancer, inflammation, and immune regulation. Investigation of the pharmacological characteristics of the EP3 receptor demonstrated smooth muscle contractility [157]. EP3 receptors mediate vasoconstriction in human arteries, including PAs [158], and are also implicated in mediating the pulmonary vascular contractions brought on by isoprostanes, which are oxidative polyunsaturated fatty acid metabolites that are significantly elevated in patients with PH [159, 160]. Five of the ten different splicing variants of EP3 (EP3-1a, EP3-1b, EP3-1c, EP3-4, and EP3-5) were markedly elevated in human PA (hPA) exposed to 1% O_2_ for 24 h [161]. EP3 expression increased in the PAs of hypoxia-induced mice and MCT-treated rats compared to normoxia mice and control rats, respectively [161, 162]. By the inhibition of Rho-dependent extracellular MMP-2/TGF-β1 signaling, disruption of EP3 improved pulmonary vascular remodeling and alleviated both hypoxia-induced and hypoxia/SU5416-induced PAH in mice. More significantly, therapy with an EP3 antagonist L-798,106 suppressed the progression of PAH in the MCT rat model [161]. In vitro or in vivo, PASMC proliferation or hypertrophy in animals with PAH is not significantly affected by EP3. In summary, overexpression of the EP3 receptor in PAs contributes to pulmonary vascular remodeling in PAH.

### Prostaglandin E_2_ receptor 4

The PTGER4 gene encodes prostaglandin E2 receptor 4 (EP4), the prostaglandin receptor of human PGE_2_, located at the p13.1 position of human chromosome 5 and containing seven exons encoding GPCRs of the rhodopsin-like receptor family [163]. EP4 is broadly expressed in human tissues [27] and has a high expression level in the gastrointestinal tract, uterus, hematopoietic tissues, and skin [164]. The relaxation of the pulmonary veins is mediated by EP4 receptors, although PAs are unaffected [165]. Bradykinin stimulates the expression of COX-2 in human PASMCs by activating the cAMP response element with EP4 and EP2 agonists [166]. Compared to the control group, IP receptor expression was decreased in the lung samples of patients with idiopathic PAH and in the lungs of MCT-induced 28d rats, but the expression of the EP4 receptor was stable [167]. An EP4 receptor antagonist, AH23848, dose-dependently decreased the rise of cytoplasmic cAMP caused by iloprost in PASMCs, but AH6809 (an EP2 receptor antagonist) had no such effect [167]. This finding indicated that under the condition of lower IP receptor expression related to PAH, iloprost exerts vasodilatory activities via the EP4 receptor. Another study reported that in the presence of lower IP expression related to PAH, the EP4-PKA-PPARγ signaling pathway exerts a significant regulatory role in suppressing PASMC proliferation and migration. L-902,688, an EP4 agonist, was effective in severe experimental PAH rodent models by raising PPARγ expression [168]. Furthermore, EP4-specific agonist L-902,688 reduced right ventricular [93] fibrosis and prevented TGF-β1-induced endothelial-mesenchymal transition (EndMT) [169]. Therefore, EP4 is a potential therapeutic target for improving pulmonary vascular remodeling and enhancing RV function of lower IP expression related to PAH. The beneficial effects of another PGI_2_ analog, beraprost (BPS), on vascular contraction in PH may be mediated, partly through interacting with the EP4 receptor and reactivating Kv channels [170]. The expression and functional reduction of Kv channels are included in the pathogenesis of hypoxia-induced PH, ultimately causing vascular remodeling and pulmonary vasoconstriction, and Kv channel upregulation has therapeutic value for PH [171]. In addition to its benefits in patients with Group 1 PAH, EP4 is also beneficial in patients with Group 3 PH with respiratory diseases. EP4 agonists can improve outcomes in patients with Group 3 PH by decreasing dyspnea and improving the capacity to perform physical effort (6 MWD).

Together, EP2 and EP4 have vasodilatory effects, while EP1 and EP3 contribute to vasoconstriction. The function of PGE_2_ on PH is complex, depending on the dominant receptor subtype at that time. Thus, selective targeting PGE_2_ receptors or combination of PGE_2_ with EP1 and EP3 inhibitors may be a promising treatment for patients with PH.

## Prostaglandin F_2α_ and prostaglandin F receptors

### Prostaglandin F_2α_

PGF_2α_ plays a significant role in the female reproductive system, and participates in physiological processes such as pregnancy physiology, the onset of labor, and postpartum uterine contraction [172]. PGF_2α_ is known to be a potent vasoconstrictor derived from the prostanoids family [173]. It is associated with hypertrophic growth of cardiomyocytes, vascular smooth muscle cells, and skeletal muscle cells [174]. ROS is required for PGF_2α_-associated vascular smooth muscle hypertrophy [175]. An analog of prostaglandin F2, 15-F2t-isoprostane (15-F2t-IsoP) mediates the vasoconstriction of PAs and resistance microvessels, as well as mitogenesis in VSMCs [176]. 15-F2t-IsoP stimulates endothelial cell proliferation and ET-1 synthesis in PAs [177]. Patients with PH have been observed to have higher urine concentrations of 15-F2t-IsoP and it is associated with survival [160]. One study reported elevated plasma concentrations of 15-F2t-isoP in 80 patients with IPAH. Moreover, individuals with baseline plasma concentrations of 15-F2t-isoP > 97 pg/ml had a considerably reduced chance of surviving, and that concentrations were elevated in patients who died during follow-up (30 ± 12 months) and reduced in those who survived [178]. It has been reported that 15-F2t-IsoP acts through the TP receptor [179].

### Prostaglandin F receptor

PGF_2α_ biological effects are mediated by FP, which comprises seven exons and is encoded by the PTGFR gene, which is found on human chromosome 1 at location p31.1 and belongs to the family of GPCRs called rhodopsin-like receptors [180, 181]. FPA and FPB are different C-terminal length isoforms of PTGFR encoded by alternatively spliced transcripts [158, 182]. FP receptors are widely distributed in human tissues and have important functions in reproduction, the central nervous system, kidneys, eyes, cardiovascular system, cancer, and bone. The systolic blood pressure of FP knockout mice was significantly lower compared to that of wild-type mice, and this was accompanied by significant reductions in plasma concentrations of renin and angiotensin-1 [183]. However, there is a lack of research on the role and function of FP in PH, necessitating further in vivo and in vitro studies in this area.

## Prostaglandin I_2_ and prostaglandin I_2_ receptor

### Prostaglandin I_2_

PGI_2_ is mostly generated by endothelial cells and VSMCs [184, 185], and has various pharmacological effects including vasodilation, inhibition of smooth muscle cell proliferation, and platelet aggregation [186]. PGI_2_ and some of its analogs are PPARα and PPARβ/δ ligands, as are some prostaglandin receptors. It is no longer possible to conceptualize PGI_2_ as a hormone that only acts biologically by activating the IP receptor [187]. PPARs are members of the nuclear receptor superfamily, and three of them (PPARα, PPARβ/δ, and PPARγ) have been identified in mammalian cells [188]. Pretreatment of human umbilical vein endothelial cells (HUVEC) with PGI_2_ prevented H_2_O_2_-induced apoptosis, while suppression of PPARβ/δ eliminated the anti-apoptotic effect of PGI_2_ [189].PGI_2_ was found to protect vascular smooth muscle cells from oxidative-induced apoptosis, although its anti-apoptotic effect was eliminated by PPARα inhibitors [190]. Therefore, PGI_2_ may exert its effects on PAH through the PPAR pathway. Prostacyclin analogs and prostacyclin receptor agonists are well established in the treatment of PAH [191]. Currently, prostacyclin analogs and prostacyclin receptor agonists are treatment options for PAH and are recommended for patients with functional Grade II–IV PAH based on the latest PAH treatment recommendations [16]. When monotherapy and other treatments fail to control symptoms in patients with PAH, these analogs are taken into consideration for combination therapy. Moreover, different prostacyclin analogs and prostacyclin receptor agonists and their preparations can be administered intravenously, subcutaneously, or via inhalation to patients with PAH. Prostacyclin analogs include iloprost, treprostinil, and beraprost. Selexipag is a prostacyclin receptor agonist. Epoprostenol, a synthetic prostacyclin, mainly acts on IP, EP3, primarily inducing vasodilation and inhibiting platelet aggregation [192]. It has been shown to significantly improve dyspnea, fatigue symptoms, and exercise capacity in patients with PAH, as well as improve hemodynamic parameters including PVR, mPAP, and cardiac index [193, 194]. Due to its short half-life (2–3 min), epoprostenol can only be administered through continuous intravenous infusion [195], which poses a risk of infection. Iloprost, acts on mainly IP, EP1, EP3, is a vasodilator and also has antiplatelet properties [196]. Inhaled iloprost improves PVR, mPAP, and 6MWD in patients with PAH [197, 198]. Treprostinil, a prostacyclin analog, acts on mainly IP, EP2, DP1, and also exerting vasodilatory effects and inhibiting platelet aggregation [199, 200]. Treprostinil has a prolonged half-life (4 h) and can be administered via intravenous, subcutaneous, inhalation, or oral routes [16]. Treprostinil administered via the intravenous, subcutaneous, or inhalation route can also improve PVR, mPAP, and 6MWD in patients with PAH [201–203]. Beraprost, a prostacyclin analog, mainly acts on IP, has vascular dilatation and antiplatelet effect [204]. Oral prostaglandin can improve PVR, mPAP, and 6MWD in patients with PAH [205, 206]. The therapeutic indications, administration, side effects, and corresponding receptors of prostacyclin analogs and prostacyclin receptor agonists are shown in Table 2. Each drug has a different affinity for the prostaglandin receptors (Table 3), which may be related to their effects.

### Prostaglandin I_2_ receptor

The prostacyclin receptor, also known as the prostaglandin I_2_ receptor or IP, modulates the biological activity of prostacyclins or PGI_2_ by binding to them. The PTGIR gene in humans encodes the IP receptor, being located at locus q13.32 on human chromosome 19 and contains six exons encoding GPCRs of the rhodopsin-like receptor family [207, 208]. Northern blot analysis revealed that the expression level of IP receptor mRNA was highest in the thymus, and high levels of IP mRNA expression were found in the lung, heart, and spleen [208, 209]. Activation of IP receptors triggers the formation of intracellular cyclic adenosine phosphate and activates protein kinase A, which mediates pulmonary artery vasodilation, inhibits platelet aggregation, and relaxes smooth muscles [210]. A knockout mouse model of the IP receptor confirmed the blood pressure-lowering and anti-aggregatory capabilities of cicaprost, as well as its anti-proliferative activities in cultured mouse PASMCs [211]. In addition to its function in vascular dilatation, proliferation inhibition, and pulmonary remodeling protection, it has been reported that PGI_2_ regulates the immune response through IP signaling [212], and also by promoting Th17 differentiation in vivo, which may have clinical significance for the application of PGI_2_ and its analogs in the treatment of PAH [213]. Selexipag, a prostacyclin receptor agonist, selectively binds to IP, can improve the prognosis of patients with PAH [214, 215]. Ralinepag is another prostacyclin receptor agonist, and a Phase 3 study for Ralinepag is ongoing. In the Phase 2 trial, Ralinepag dramatically decreased PVR in patients with PAH [216].

## Thromboxane A_2_ and thromboxane A_2_ receptor

### Thromboxane A_2_

TXA_2_ is mainly produced by platelets, lung, kidney, and intestinal parenchymal cells, and has various pharmacological effects, including platelet aggregation, airway constriction, and contraction of different types of arteries, including PAs [38]. In vitro, TXA_2_ pretreatment significantly promotes hypoxic pulmonary vasoconstriction. In vivo, administration of TXA_2_-mimicking drug U46619 caused pulmonary vasoconstriction, leading to an increase in PVR and mPAP, decrease in CO, and U46619 can be utilized to construct PH in dogs [217, 218]. In addition, TXA_2_ production and disruption of TP signaling attenuated the detrimental impact on pulmonary and cardiac metrics in a porcine hypoxia-induced PH model [219]. TXA_2_ exerts its pharmacological effects via the TP receptor.

### Thromboxane A_2_ receptor

The TBXA2R gene, which is found on chromosome 19 at location p13.3, encodes TP, generally referred to as the TXA_2_ receptor [220]. TBXA2R encodes a member of the G protein-coupled superfamily of seven-transmembrane receptors [221]. There are two subtypes of human TP receptor: TPα and TPβ [222]. Platelets exhibit significant levels of the α isoform, but the β isoform is not identified [221]. The β isoform is generated in human endothelial cells. Rodents exhibit the TPα isoform only. These rodents are utilized as animal models to clarify how genes and their byproducts work; however, they do not have two TP isoforms, which limits our recognizing of the various roles played by each TP receptor isoform [158]. Research has mainly focused on the role of TP receptors in platelet function. Nonetheless, it is now evident that TP receptors are widely distributed in various systems and cells types [221]. TP receptors have been identified in heart, immunological, reproductive, lung, and nervous system tissues [27]. Several studies have demonstrated the significance of TP signaling in the onset and development of PH [223–227]. In a porcine model, the TP antagonist daltroban attenuated hypoxic pulmonary vasoconstriction by decreasing mPAP [219]. In rats, TP receptor activation induces a contractile response through Rho kinase signal [228]. NTP42, an antagonist of TP, attenuated the MCT-induced PAH, comparable effectiveness to that of Selexipag or Sildenafil, which are the standard treatments. In addition, in MCT-treated rats, NTP42 significantly improved pulmonary vascular remodeling, inflammatory mast cell infiltration, and fibrosis with greater effects than those observed with Sildenafil and Selexipag [229]. Furthermore, NTP42:KVA4, an oral formulation of NTP42, was found to alleviate pulmonary pathologies, reduce right ventricular remodeling, and improve hypertrophy, by antagonizing TP signaling, thereby reducing PAH pathophysiology and improving cardiac function [230]. Moreover, the elevations in mPAP and right systolic ventricular pressure (RSVP) induced by hypoxia/SU5416 was significantly decreased by the combination of NTP42 and Sildenafil; however, Sildenafil or NTP42 mono-therapy did not reduce the increase in mPAP and RSVP. Comprehensive histological analyses suggested that combined treatment with NTP42 and Sildenafil was considerably more beneficial for pulmonary vessel remodeling, right ventricular hypertrophy, and fibrosis than monotherapy with either drug alone. Sildenafil exhibits vasodilatory and anti-proliferative effects by NO signaling pathway, while NTP42 effectively inhibits excessive vasoconstriction and microvascular thrombosis by antagonizing TP signaling. Both NTP42 and Sildenafil contribute to the reduction of PH through distinct yet complementary mechanisms [231]. It is considerably more beneficial to treat or counteract the main etiologies underlying PAH when NTP42 and Sildenafil are used together in dual treatment [231]. Increased availability of TXA_2_ as well as increased contractile sensitivity to TXA_2_ may increase pulmonary circulation burden in PAH. The thickening of pulmonary arterial walls increases right ventricular afterload and peripheral resistance with functional changes in PAH [38]. Therefore, TP plays an important role in PAH and its progression and can influence right heart function. This is a promising intervention target for PAH.

## Conclusions

**Fig. 5:**
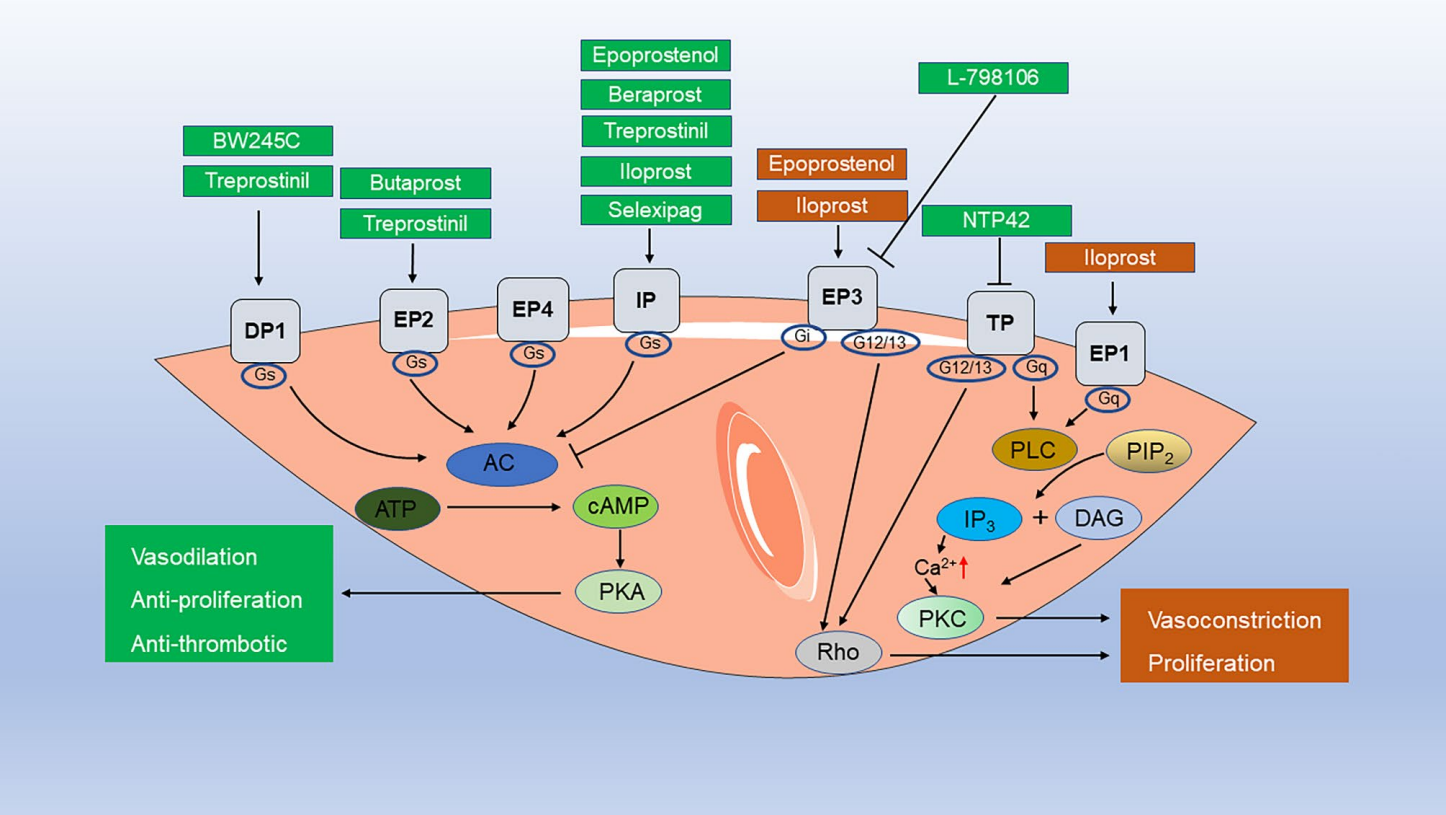
Prostacyclin drugs and prostaglandin receptor agonists/antagonists are involved in the pathogenesis of pulmonary arterial hypertension (PAH) through acting on distinct prostaglandin receptors. DP1 (activated by BW245C, treprostinil), EP2 (activated by butaprost, treprostinil), EP4, IP (activated by epoprostenol, beraprost, treprostinil, iloprost, selexipag) promoted vasodilation, anti-proliferation, and anti-thrombotic effects through the cAMP signaling pathway. EP1 (activated by iloprost), EP3 (activated by epoprostenol, iloprost, inhibited by L-798,106), TP (inhibited by NTP42) promoted vasoconstriction and proliferation through Rho and PKC signaling pathways. BW245C, a DP1-specific agonist; Butaprost, a highly selective EP2 receptor agonist; L-798,106, an EP3 antagonist; NTP42, a TP antagonist. Abbreviation: AC, adenylate cyclase; ATP, adenosine triphosphate; cAMP, cyclic adenosine monophosphate; DAG, diacylglycerol; IP3, inositol triphosphate; PIP2, phosphatidylinositol (4,5) bisphosphate;PKA, protein kinase A; PKC, protein kinase C; PLC, phospholipase C

In this review, we investigated the role of the PGs pathway in the occurrence and development of PAH (Table 4). PGs exert their effects on pulmonary vascular remodeling by mediating the proliferation and hypertrophy of PVSMCs, endothelial cells, and other cell types. PGs are essential PAH mediators and, to date, research has focused on identifying the signaling pathways that PGs receptors activate in PAH. Different receptors have different physiological effects: DP1, EP2, EP4, and IP signaling pathways mainly improve pulmonary vascular remodeling and thus improve PAH (Fig. 3), while DP2, EP3, and TP signaling pathways aggravate PAH (Fig. 4). In the extracellular microenvironment, PGs bind to GPCRs and influence PAH development. We further summarized the potential drugs targeting PGs and their receptors, which might be involved in PAH (Fig. 5). Prostacyclin analogs and prostacyclin receptor agonists have effectively improved the quality of life of patients with PAH, although they have not been able to inhibit or reverse pulmonary vascular remodeling [232]. The mortality rate of patients with PH is up to 10% in one year and has not declined [233]. Prostacyclin and prostacyclin analogs, including epoprostenol, beraprost, iloprost, and treprostinil, do not act solely on the IP receptors. Epoprostenol mainly acts on IP and EP3; beraprost mainly acts on IP; iloprost mainly act on IP, EP1and EP3; and treprostinil mainly acts on IP, EP2, and DP1. Since the activation of EP3 receptors can aggravate PAH, whether their effect on EP3 receptors has any clinical effect remains unknown. However, combination therapy with EP3 receptor antagonists has not yet been reported. The treatment for patients with PAH is heterogeneous, and more targeted treatment is needed. Selexipag acts specifically on IP receptors. However, studies have shown no significant differences in 6 MWD, PVR, mortality, or adverse events compared with the control groups [234]. Thus, the specific binding of IP receptors does not reduce adverse events or mortality in patients with PAH. PGI_2_ has been shown to function via the PPAR signaling pathway. The presence of other receptors for prostacyclin analogs and prostacyclin receptor agonists remains unknown. Furthermore, the downstream signaling pathways of prostaglandin receptors are promising targets for PAH therapy. Everolimus, an mTOR inhibitor, improves the 6MWD and PVR in patients with PAH [235]. Fasudil, a Rho kinase inhibitor, is effective and safe for improving mPAP, PVR, and CI in patients with PAH in the short- and medium-term [236]. Combination therapy with prostaglandin receptor agonists, antagonists, or drugs targeting downstream sites may improve PAH. As, evidenced by many of the studies in this review, most of the evidence for the role of PGs receptors comes from in vitro or animal studies; clinical transformation is slow. In particular, the roles of EP1 and FP in PH remain unclear. Existing medical therapies primarily target Group 1 PH (PAH). Moreover, treprostinil has been shown to improve 6MWD in patients with Group 3 PH (PH associated with interstitial lung disease) and Group 4 PH (chronic thromboembolic PH) [237, 238]. Further research is needed to investigate the therapeutic effects of prostaglandins and related drugs on different groups of PH. In summary, we examined the function of PGs signaling pathways in PAH and suggest that targeting PGs pathways may provide opportunities for PAH prevention and treatment.

**Table 4:** A summarization of prostaglandin receptors signaling in PH

Targets	Functional cell types	Mechanism	Phenotypic features
DP1	VSMCs	cAMP/PKA	Vasodilation [27]
	PASMCs	PKA/raptor-dependent mTORC1 dissociation	Inhibit proliferation and hypertrophy [49]
DP2	Th2	Release IL-4, IL-13	Promote PASMCs proliferation by activating STAT6 [113]
EP1	VSMCs	PLC/PKC	Vasoconstriction [27]
EP2	VSMCs	cAMP/PKA	Vasodilation [27]
EP3	VSMCs	Inhibit cAMP/PKA	Vasoconstriction [27]
	PASMCs	Rho-dependent extracellular MMP-2/TGF-β1	Extracellular matrix remodeling [161]
EP4	VSMCs	cAMP/PKA	Vasodilation [166]
	PASMCs	PKA/PPARγ	Inhibit PASMCs proliferation and migration [168]
	PASMCs	Kv channels	Vasodilation [170]
IP	VSMCs	cAMP/PKA	Vasodilation [187]
TP	VSMCs	PLC/PKC	Vasoconstriction [27]
	PASMCs	Rho	Vasoconstriction [228]
	PASMCs	Inhibit Kv channels	Inhibit proliferation [230]

## Data Availability

Not applicable.

## Abbreviations

12-HETE: 12-hydroxy-5,8,10,14-eicosatetraenoic acid
15-F2t-IsoP: 15-F2t-isoprostane
6MWD: Six-minute walk distance
AA: Arachidonic acid
AC: Adenylate cyclase
ATP: Adenosine triphosphate
BMEPCs: Bone marine-derived endothelial progenitor cells
cAMP: Cyclic adenosine monophosphate
CO: Cardiac output
COX: Cyclooxygenase
Cpc PH: Combined post- and pre-capillary pulmonary hypertension
CREB: cAMP-response element binding protein
CRTH2: Chemoattractant receptor homologous molecule expressed on Th2
CYP: Cytochrome P450
DAG: Diacylglycerol
DEGs: Differentially expressed genes
DP1: PGD2 receptor 1
DP2: PGD2 receptor 2
EETs: Epoxyeicosatrienoic acids
EP1: PGE2 receptor 1
EP2: PGE2 receptor 2
EP3: PGE2 receptor 3
EP4: PGE2 receptor 4
ET-1: Endothelin-1
FP: Prostaglandin F receptor
GEF: Guanine nucleotide exchange factor
GPCR: G-protein-coupled receptor
GSK-3β: Glycogen synthetase kinase 3β
HPASMCs: Human pulmonary artery smooth muscle cells
H-PGDS: Hematopoietic PGDS
HUVEC: Human umbilical vein endothelial cells
i.v.: Intravenous
ILC2: The group 2 innate lymphoid cell
IP: Prostacyclin receptor
IP3: Inositol triphosphate
IPA: Intrapulmonary artery
IRF7: Interferon regulatory factor 7
Jak: Janus kinase
LOX: Lipoxygenase
L-PGDS: Lipocalin-type PGDS
LTB4: Leukotriene B4
MCT: Monocrotaline
MMP: Matrix metalloproteinase
mPAP: Mean pulmonary arterial pressure
MRTF-A: Myocardin-related transcription factor A
MT1-MMP: Membrane type 1-matrix metalloproteinase
mTORC1: Mammalian target of rapamycin complex 1
NF-κB: Nuclear factor κB
NO: Nitric oxide
PAECs: Pulmonary artery endothelial cells
PAH: Pulmonary arterial hypertension
PAP: Pulmonary arterial pressure
PAs: Pulmonary arteries
PASMCs: Pulmonary artery smooth muscle cells
PGD2: Prostaglandin D2
PGDS: Prostaglandin D synthase
PGE2: Prostaglandin E2
PGES: Prostaglandin E synthase
PGF2α: Prostaglandin F2α
PGFS: Prostaglandin F synthase
PGHS: Prostaglandin H synthase
PGI2: Prostaglandin I2
PGIS: Prostaglandin I synthase
PGs: Prostaglandins
PH: Pulmonary hypertension
PI3K: Phosphoinositide 3-kinase
PIP2: Phosphatidylinositol (4,5) bisphosphate
PKB: Protein kinase B
PKC: Protein kinase C
PLA2: Phospholipase A2
PLC: Phospholipase C
PPAR: Peroxisome proliferator-activated receptor
PVR: Pulmonary vascular resistance
PVSMCs: Pulmonary vein smooth muscle cells
Rock: Rho-associated coiled-coil-containing protein kinase
RSVP: Right systolic ventricular pressure
SERT: Serotonin
SMCs: Smooth muscle cells
SOCC: Store-operated calcium channels
STAT6: Signal transducer and activator of transcription 6
TGF-β1: Transforming growth factor β1
TP: TXA2 receptor
TXA2: Thromboxane A2
TXAS: Thromboxane A synthase
VEGFR-2: The VEGF receptor-2
VOCC: Voltage-operated calcium channels
vWF: Von Willebrand factor
